# Supplementary material for: Fabrication of Robust Superhydrophobic Surfaces with Dual-Curing Siloxane Resin and Controlled Dispersion of Nanoparticles
Source: Polymers (Basel). 2020 Jun 25;12(6):1420. doi: 10.3390/polym12061420 (PMC7362197; doi:10.3390/polym12061420)
Supplement: Supplementary file 1 [file polymers-12-01420-s001.zip › polymers-829385-supplementary.docx]

Supplementary Information

Fabrication of Robust Superhydrophobic Surfaces with Dual-Curing Siloxane Resin and Controlled Dispersion of Nanoparticles

Hyeran Kim, Kibeom Nam and Dong Yun Lee*

Department of Polymer Science and Engineering, Kyungpook National University, Daegu 41566, Republic of Korea; [rwnka110@naver.com](mailto:rwnka110@naver.com) (H.K.); [ska2918@naver.com](mailto:ska2918@naver.com) (K.N.)

***** Correspondence: E-mail: dongyunlee@knu.ac.kr, Tel: +82 53 950 5627, Fax: +82 53 950 6623 (D.Y.L.)

**
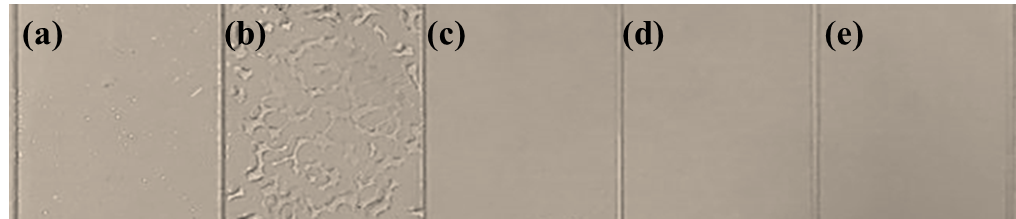
**

**Figure S1.** Photographs of spin-coated samples prepared with GOTMS:APTES molar ratios of (a) 1:0.1, (b) 1:0.25, (c) 1:0.5, (d) 1:1, and (e) 1:2.

**Table S1.** DI water and diiodomethane contact angles and calculated surface energies.

| **FAS content (mol%)** | **0.0** | **1.0** | **1.3** | **2.0** | **4.0** | **6.0** | **10.0** |
| --- | --- | --- | --- | --- | --- | --- | --- |
| **DI water CA (degree)** | **59** | **97** | **99** | **105** | **107** | **107** | **108** |
| **Diiodomethane CA (degree)** | **29** | **69** | **72** | **77** | **78** | **77** | **78** |
| **Surface energy (mJ/m^2^)** | **55.5** | **24.7** | **22.8** | **19.8** | **18.9** | **19.2** | **18.7** |

**
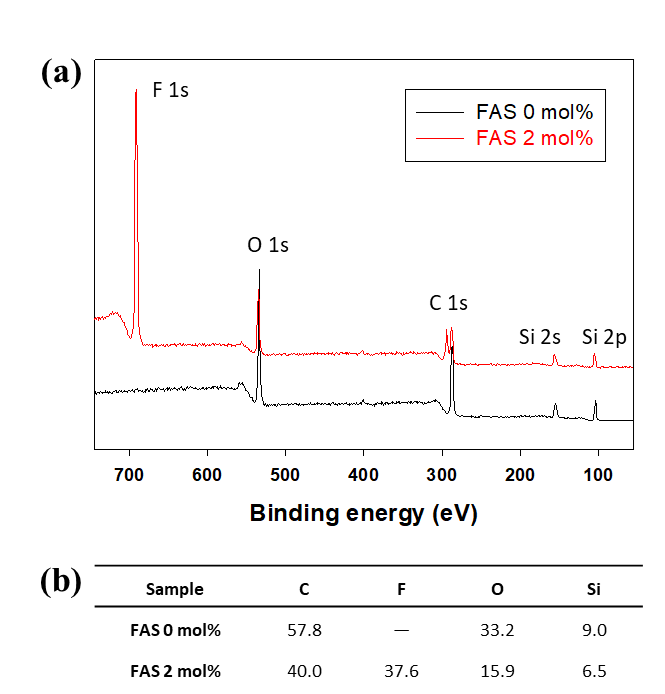
**

**Figure S2.** (a) XPS spectra of spin-coated siloxane resins with FAS contents of 0 and 2 mol% on glass substrates. (b) XPS elemental analysis of spin-coated siloxane resins with FAS contents of 0 and 2 mol% on glass substrates.

**Table S2.** Pencil hardness of spray-coated surfaces obtained under different coating conditions. Variables that were kept constant included the sonication time (5 min), NP content (1.0 wt%), and binder content (5 wt%).

| **Sonication time (min)** | **1** | | **5** | | **10** | | **30** | | **60** | | **90** | |
| --- | --- | --- | --- | --- | --- | --- | --- | --- | --- | --- | --- | --- |
| **Hardness** | **B–HB** | | **H–2H** | | **2H–3H** | | **6H–7H** | | **> 9H** | | **> 9H** | |
|  |  | |  | |  | |  | |  | |  | |
| **NP content (wt%)** | **0.5** | | **0.7** | | **1.0** | | **1.5** | | **2.0** | | **4.0** | |
| **Hardness** | **5H–6H** | | **3H–4H** | | **H–2H** | | **B** | | **5B** | | **< 6B** | |
|  |  | |  | |  | |  | |  | |  | |
| **Binder content (wt%)** | | **1** | | **3** | | **5** | | **7** | | **10** | |  |
| **Hardness** | | **6B** | | **2B–3B** | | **H–2H** | | **6H** | | **9H** | |  |


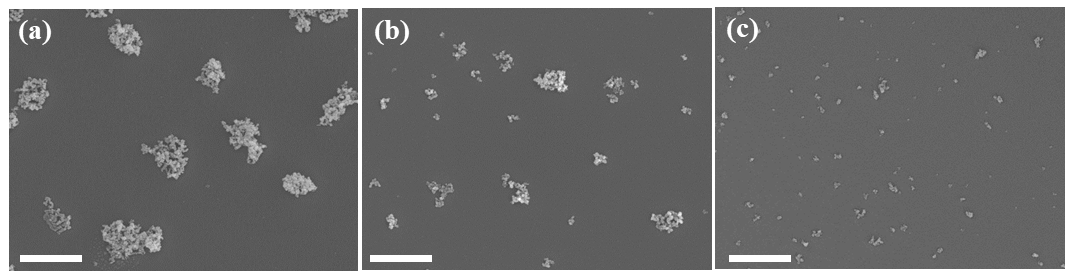


**Figure S3.** SEM images of NPs after sonication for (a) 1, (b) 30, and (c) 90 min. Scale bar=1 μm.

**
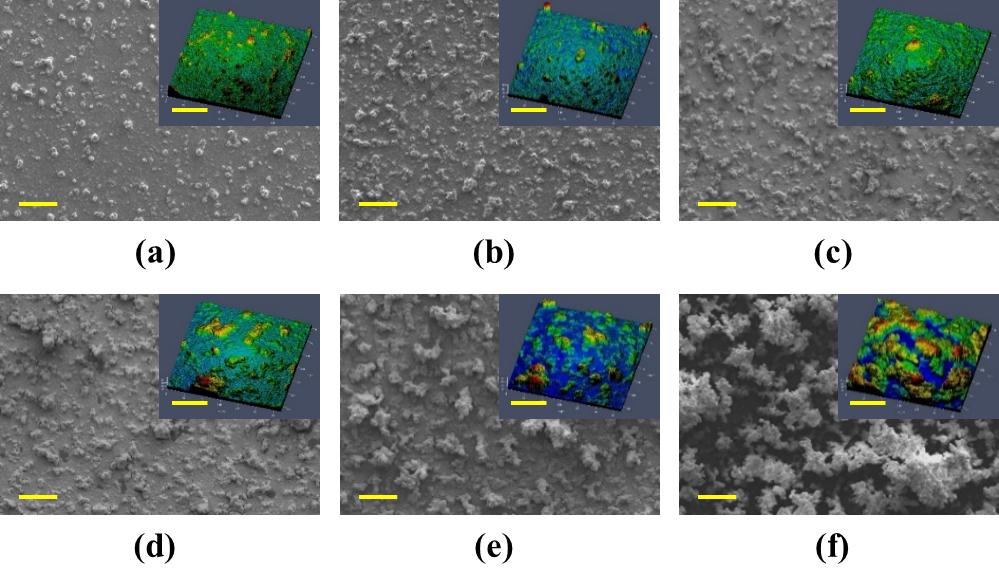
**

**Figure S4.** SEM and confocal laser scanning microscope images of spray-deposited NPs on Si wafers at NP concentrations of (a) 0.5, (b) 0.7, (c) 1.0, (d) 1.5, (e) 2.0, and (f) 4.0 wt%. Scale bar = 100 μm.


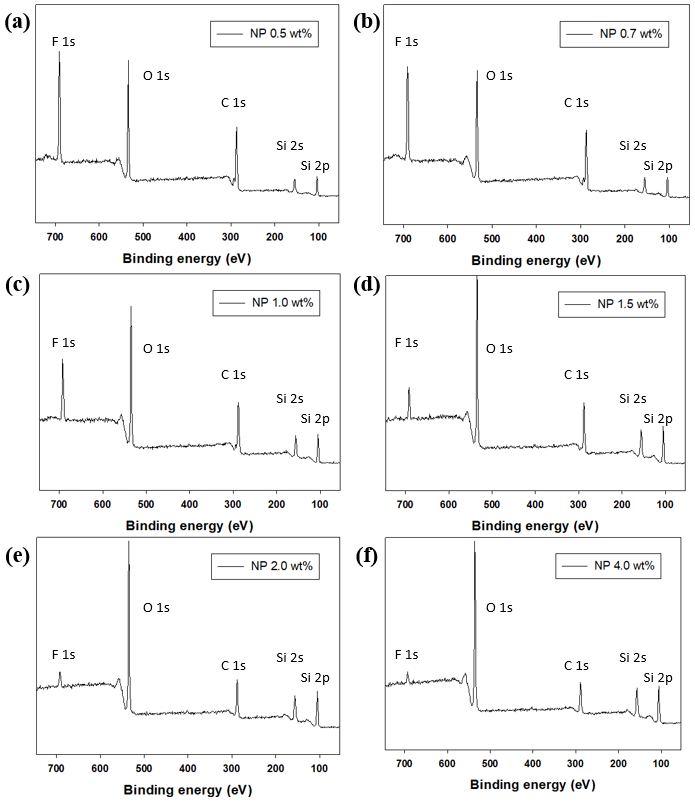


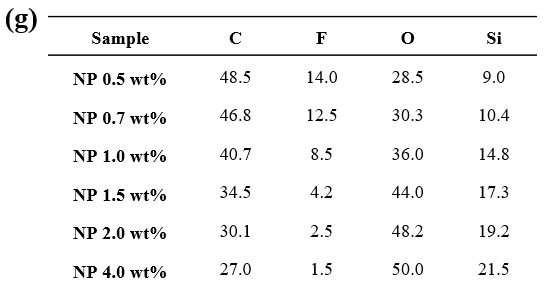


**Figure S5.** XPS spectra of coatings with NP contents of (a) 0.5, (b) 0.7, (c) 1.0, (d) 1.5, (e) 2.0, and (f) 4.0 wt%. (g) Elemental compositions of the samples.


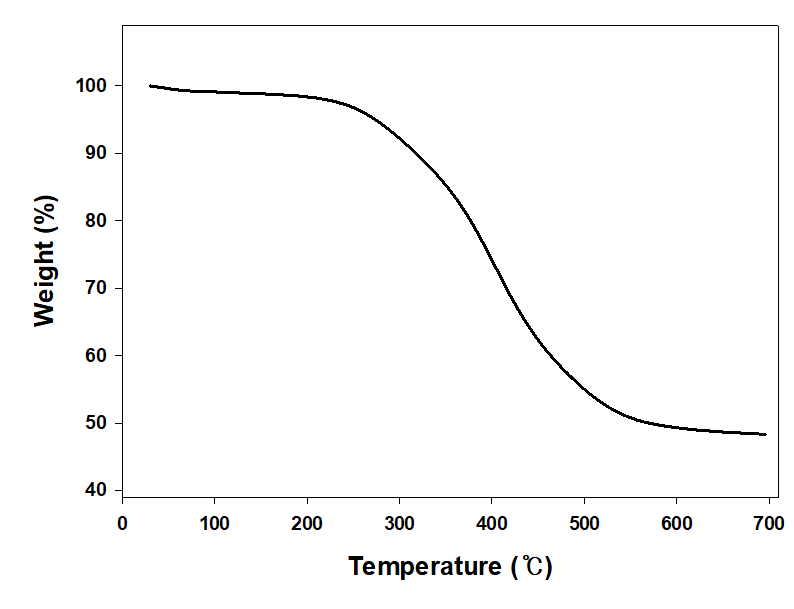


**Figure S6.** TGA curve of the siloxane resin in a nitrogen atmosphere.


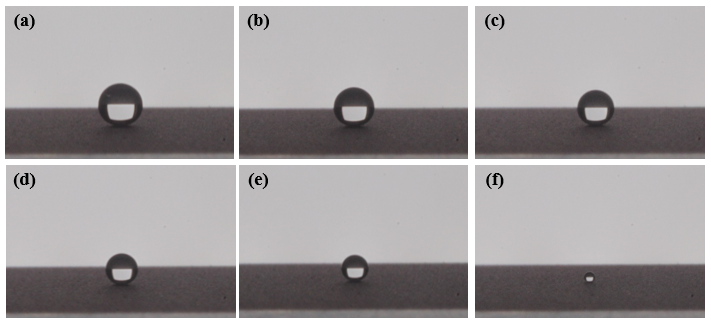
**Figure S7.** Photographs of CAs changes while staying water droplets on the superhydrophobic surface for (a) 0, (b) 5, (c) 10, (d) 15, and (e) 20, and (f) 30 min.

**Movie S1.** Drops of water dyed with methylene blue on superhydrophobic surfaces tilted at 15°,

**Movie S2.** Drops of water dyed with methylene blue on patterned superhydrophobic surfaces tilted at 15°
